# Supplementary material for: The Effect of Phosphate on the Activity and Sensitivity of Nutritropism toward Ammonium in Rice Roots
Source: Plants (Basel). 2022 Mar 9;11(6):733. doi: 10.3390/plants11060733 (PMC8955032; doi:10.3390/plants11060733)
Supplement: Supplementary file 1 [file plants-11-00733-s001.zip › Supplementary files/Supplementary Figure S1.pdf]

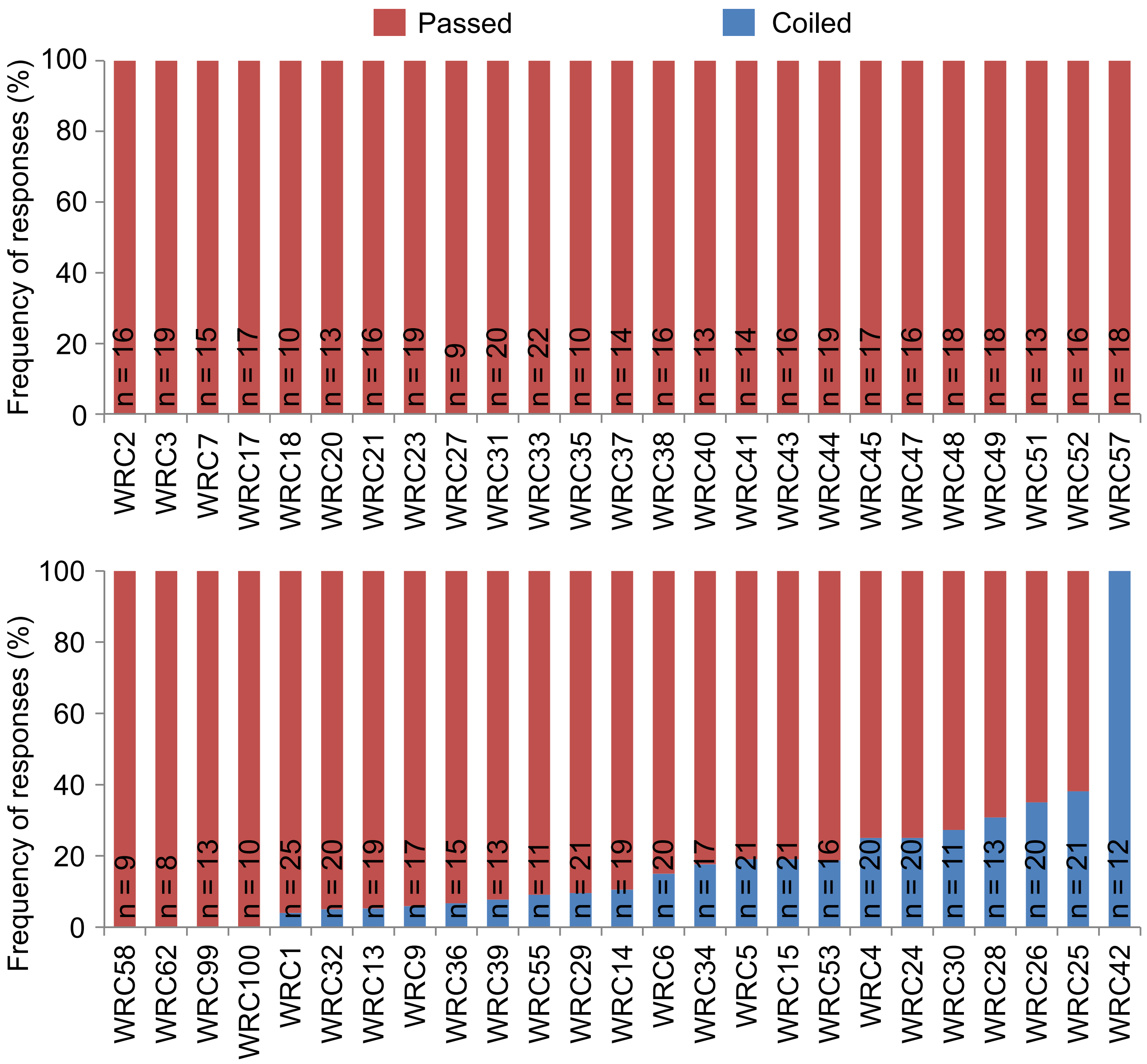

Supplementary Figure S1. Nutritropic responses of main roots (passed or coiled) of WRCs. Frequencies of passed and coiled responses were determined in the nutritropic bioassay with nutrient sources containing 200 mM NH_4^+, NO_3^+, K^+, and P*i*.
