# Supplementary material for: The Effect of Phosphate on the Activity and Sensitivity of Nutritropism toward Ammonium in Rice Roots
Source: Plants (Basel). 2022 Mar 9;11(6):733. doi: 10.3390/plants11060733 (PMC8955032; doi:10.3390/plants11060733)
Supplement: Supplementary file 1 [file plants-11-00733-s001.zip › Supplementary files/Supplementary Figure S2.pdf]

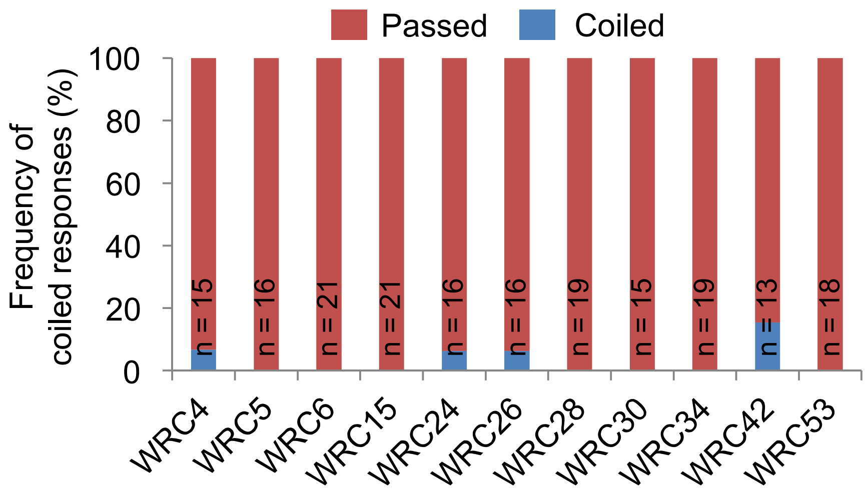

Supplementary Figure S2. Nutritropic responses of main roots (passed or coiled) of the top 11 accessions of WRCs showing coiled response in Supplementary Figure S1 (except WRC 25) to sole NH_4^+. Frequencies of passed and coiled responses were determined in the nutritropic bioassay with nutrient sources containing 200 mM NH_4^+.
