# Supplementary material for: The Effect of Phosphate on the Activity and Sensitivity of Nutritropism toward Ammonium in Rice Roots
Source: Plants (Basel). 2022 Mar 9;11(6):733. doi: 10.3390/plants11060733 (PMC8955032; doi:10.3390/plants11060733)
Supplement: Supplementary file 1 [file plants-11-00733-s001.zip › Supplementary files/Supplementary Figure S3.pdf]

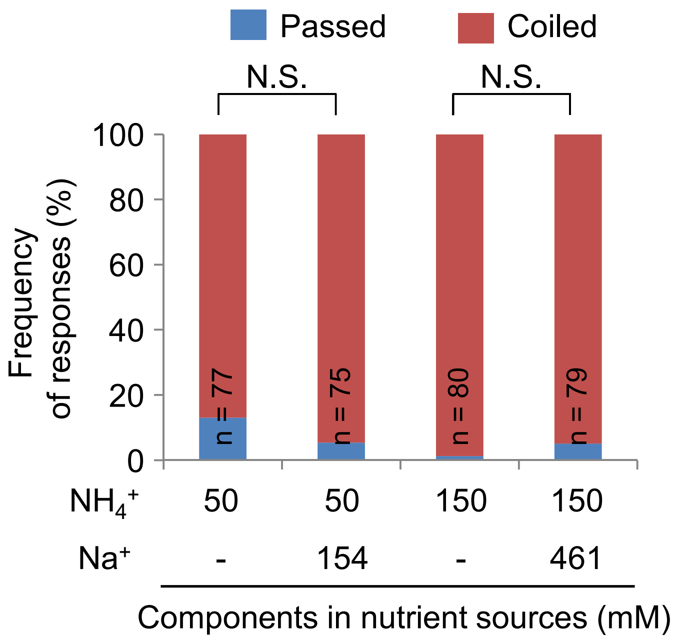

Supplementary Figure S3. Effect of Na^+ on nutritropic response of main roots (passed or coiled) of WRC 25. Frequencies of passed and coiled responses were determined in the nutritropic bioassay with 50 mM NH_4^+ + 150 mM Na^+ and with 154 mM NH_4^+ + 461 mM Na^+. The Na^+ (derived from NaCl) concentrations were adjusted to match the concentrations in 100 mM and 300 mM P*i*, where Na^+ from NaOH for neutralization and NaH_2PO_4. Statistical significance was tested using Fisher's exact test. N.S., not significant.
