# Supplementary material for: The Effect of Phosphate on the Activity and Sensitivity of Nutritropism toward Ammonium in Rice Roots
Source: Plants (Basel). 2022 Mar 9;11(6):733. doi: 10.3390/plants11060733 (PMC8955032; doi:10.3390/plants11060733)
Supplement: Supplementary file 1 [file plants-11-00733-s001.zip › Supplementary files/Supplementary Figure S4.pdf]

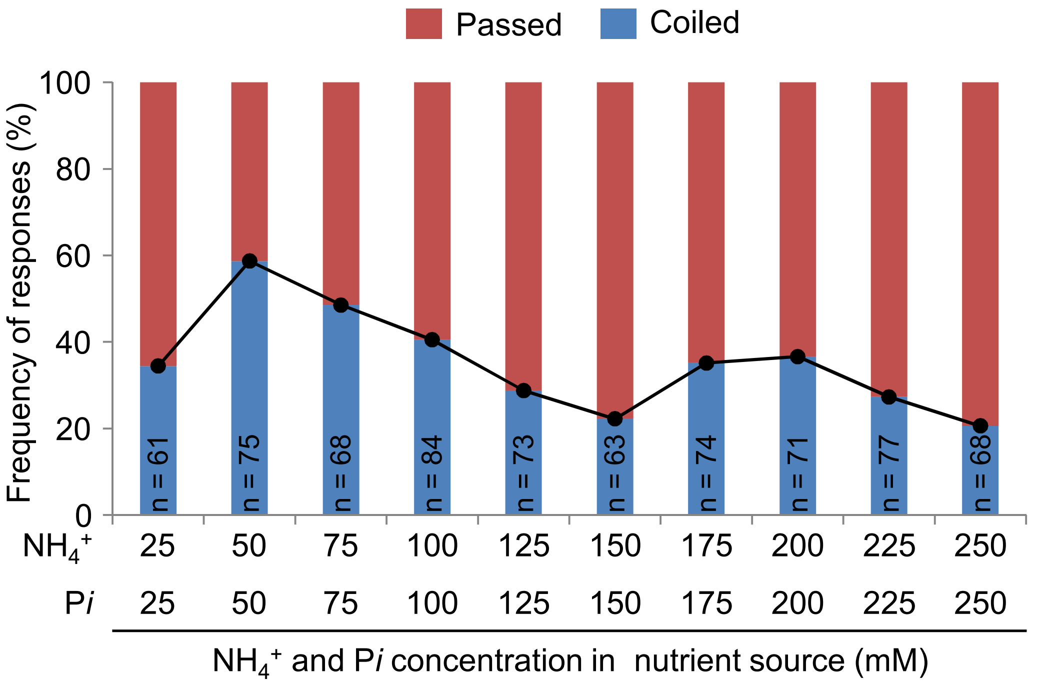

Supplementary Figure S4. Nutrient intensity response curve for nutritropic response of main roots (passed or coiled) of WRC 25. Frequencies of passed and coiled responses were determined in the nutritropic bioassays with nutrient sources containing 25, 50, 75, 100, 125, 150, 175, 200, 225 or 250 mM NH_4^+ and P*i*.
