# Supplementary material for: The Effect of Phosphate on the Activity and Sensitivity of Nutritropism toward Ammonium in Rice Roots
Source: Plants (Basel). 2022 Mar 9;11(6):733. doi: 10.3390/plants11060733 (PMC8955032; doi:10.3390/plants11060733)
Supplement: Supplementary file 1 [file plants-11-00733-s001.zip › Supplementary files/Supplementary Figure S5.pdf]

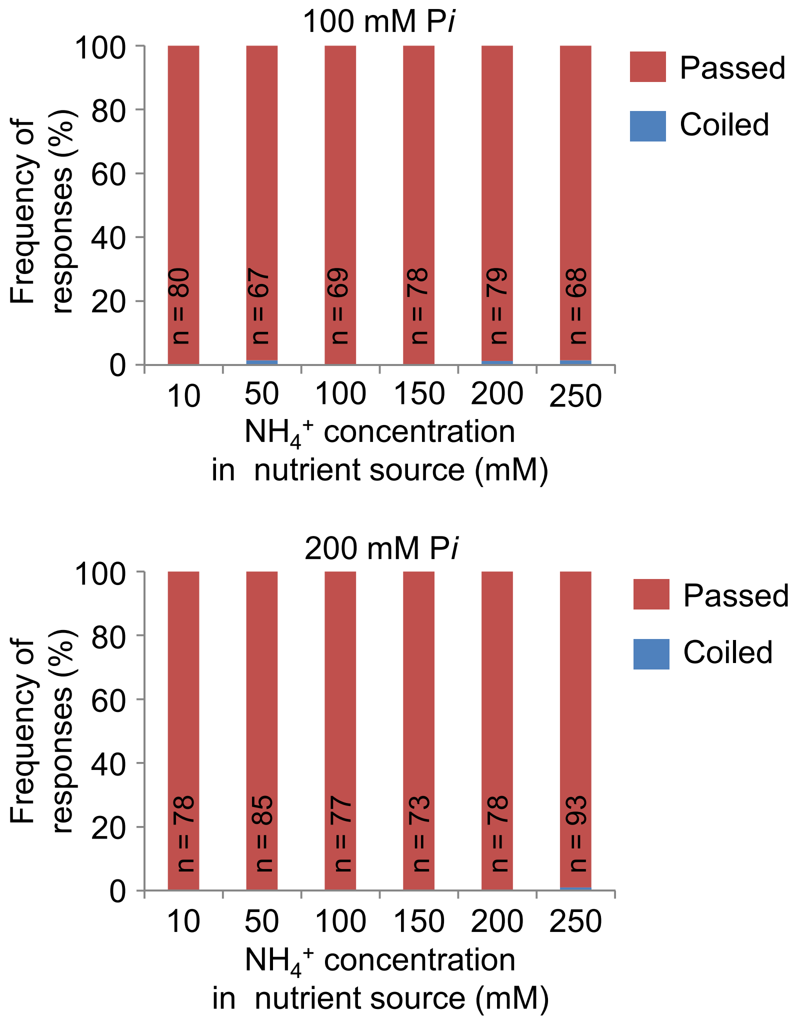

Supplementary Figure S5. Nutritropic responses of main root (passed or coiled) of WRC 1 to various concentrations of NH_4^+ and P*i*. Frequencies of passed and coiled responses were determined in the nutritropic bioassay with nutrient sources containing NH_4^+ and P*i* at the indicated concentrations.
